# Our Contemporaries

**Published:** 1918-03

**Authors:** 


### OUR CONTEMPORARIES

The Pacific Medical Journal, the oldest journal on the Pacific coast, which has just completed its 60th volume, has been acquired by Dr. William J. Robinson and will be consolidated with The American Journal of Urology and Sexology. The combined journal will continue under the editorship of Dr. Robinson and will be published from 12 Mt. Morris Park West, New York City.

The St. Paul Medical Journal has been discontinued in order to clear the field for Minnesota Medicine, the journal of the Minnesota Medical Association, affiliated with the bureau of the American Medical Association.

